# The analysis of waste heat recovery in steel enterprises’ data centers based on the Co-ah cycle

**DOI:** 10.1371/journal.pone.0323455

**Published:** 2025-05-29

**Authors:** Kai Wen, Chengjian Zhou, Xiaopo Wang

**Affiliations:** 1 Traditional Energy Services Division, Yunnan Electric Power Test and Research Institute (Group) Co., Ltd., Kunming, Yunnan, China; 2 Key Laboratory of Thermo-Fluid Science and Engineering, Ministry of Education, Xi’an Jiaotong University, Xi’an, Shaanxi, China; University of Georgia, UNITED STATES OF AMERICA

## Abstract

To improve the energy economic efficiency of Data Centers (DCs) in steel enterprises, a centralized heating scheme for waste heat recovery based on the Co-ah cycle is proposed. This scheme establishes a thermoelectric connection between the self-owned power plant of the steel enterprise and the DC, creating a waste heat recovery centralized heating system for a 15 MW DC. The energy efficiency indicators, environmental benefits, economic feasibility, and adaptability of the system are evaluated. The results show that the system can effectively recover waste heat from the DC, significantly reducing cooling electricity consumption during the heating season and decreasing original heating steam consumption by about 25%. Compared to DC using free cooling, the annual operating cost is reduced by 9.7%, with a dynamic payback period for equipment of 6–7 years. The system saves 3,671.5 tons of standard coal and reduces CO_2_ emissions by 1,615 tons annually compared to DC using isolated free cooling and traditional heating systems. The Improved Power Usage Effectiveness (PUE’) of the system is 1.195, and the Energy Reuse Effectiveness (ERE) is 0.769, outperforming the free cooling’s index of 1.341, although Exergy Reuse Effectiveness (ExRE) is slightly higher than that of free cooling. This system offers mutual benefits for self-owned power plant, DC, and heating companies, achieving a win-win operational state through suitable energy trading prices. The research conclusion provides valuable reference for the future investment and operation of DCs in steel enterprises.

## Introduction

In recent years, due to the needs for technological upgrades and industrial structural adjustments, steel enterprises have increasingly emphasized the value of Data Centers (DCs). Several medium and large-scale DCs have already been established by domestic steel enterprises, including Baosteel and Ansteel, which have operational or under-construction DCs. Predictably, as the scale of DCs in steel enterprises grows, higher energy consumption has become inevitable. Research has shown that electrical power consumption in DCs is usually converted to low-grade waste heat at a rate of about 90%, which creates opportunities for nearby various applications to utilize DCs waste heat. In addition, as the scale of DCs continues to expand, their annual energy consumption is growing rapidly, which urgently requires efficient and feasible waste heat recovery solutions [1,2]. From an energy-saving perspective, recovering waste heat from steel enterprise DCs and integrating it with self-owned power plants for energy utilization, such as centralized heating, can lead to significant savings in heating steam and achieve reductions in consumption and emissions at the system level. However, there is currently a lack of research reports on this topic both domestically and internationally.

Among the common methods for waste heat recovery in DCs, direct heating is a relatively mature technology [3–5]. Researchers have conducted in-depth studies on the organization and evaluation of DC waste heat utilization. For instance, Yu et al. explored an air-cooled DC and its auxiliary buildings in Harbin. The waste heat from the DC is used to heat the auxiliary buildings, demonstrating that this approach is more economically viable than air-source heat pumps when all DC equipment is operational [6]. Socci et al. evaluated the feasibility of integrating DC waste heat into the third-generation district heating network through high-temperature heat pumps. Real case analysis shows that the proportion of waste heat in the energy structure of district heating networks can reach 12% to 59%, which can reduce natural gas consumption by 11% to 58%. This demonstrates the enormous potential of this method in reducing the environmental footprint of cogeneration systems driven by natural gas [7]. Zou et al. proposed a DC composite energy supply system that simultaneously includes multiple cooling sources and waste heat recovery. A study on a DC in Nanchang, China shows that the Coefficient of Performance (COP) of refrigeration units in a composite energy supply system is improved by 0.4–2.55 compared to an isolated system. The COP of the heat pump during the heating season is increased by 1.4–2.1, with a maximum energy-saving potential of 72.98%, and the investment payback period is no more than 4 years [8]. Huang et al. proposed a compression assisted absorption refrigeration-heating hybrid system based on an absorption cycle. The influence of heat source parameters and compression assisted processes on system performance is explored. The results show that the compression assisted process can reduce the minimum temperature requirement for driving the heat source from 52.0°C to 27.2°C. A new path for DC waste heat recovery is developed by providing an additional 1.37 kW of heating while meeting the cooling demand of liquid cooled DC 4.29 kW [9]. Wang et al. constructed six thermodynamic models using low global warming potential refrigerants to evaluate the performance of high-temperature heat pumps for heating at 120°C. A case study is conducted on the high-performance DC waste heat recovery of US Department of Energy’s Oak Ridge National Laboratory campus and campus area heating project. For the waste heat characteristics of this DC, they recommend using the R1234ze(Z) single-stage cycle with built-in heat exchanger + economizer/parallel compressor scheme. The data shows that the 1 megawatt high-temperature heat pump system reduces carbon emissions by 33,100–33,200 tons [10]. Wahlroos et al. recovered waste heat from DCs in the district heating system in Espoo, Finland, reducing operational costs by 0.6% to 7.3%, while also improving the energy efficiency of DCs. However, the pricing of waste heat directly affects its utilization level [11]. Pärssinen et al. assessed the economic feasibility of direct heating from waste heat recovery in DCs with different sizes. The investments in small-scale waste heat utilization equipment lack reasonable economic justification, while large-scale DC waste heat recovery presents a net present value of 16.33 million euros, making it a profitable investment opportunity [12]. Nevertheless, most studies utilize electric heat pumps to meet the temperature requirements of DC waste heat, which may not always be energy-efficient or economical. Furthermore, these waste heat recovery systems are often isolated and reliant on the inherent energy resources of DCs.

Currently, there are DCs operating in conjunction with Gas-Steam Combined cycle (CCPP) and Combined Cooling, Heating, and Power (CCHP) projects. This allows DCs to achieve cooling and waste heat recovery using self-generated power, medium-low pressure steam, or high-temperature flue gas [13–15]. Davies et al. established a CCHP system that integrates a 3.5 MW wind-cooled DC in London. This demonstrates that upgrading DC waste heat with heat pumps can reduce approximately 4,000 tons of CO_2_ emissions annually, generating nearly £1 million in annual profits [16]. Huang et al. explored a cloud computing industrial park in Chongqing, which integrates numerous related enterprises and DCs. A CCPP system suitable for the park is proposed, which can recover the waste heat of condensate water and DC chilled water from the power plant, providing sufficient heat for regional heating [17]. However, the economic viability of these systems is overly dependent on the relationship between natural gas and electricity prices, and there is a lack of studies specifically analyzing waste heat recovery from steel enterprise DCs.

The Co-ah cycle, an advanced absorption waste heat recovery and centralized heating technology proposed by Fu et al., has achieved significant energy-saving effects in power plants located in Datong, Chifeng, and Yinchuan [18–20]. This technology possesses strong capabilities for low-grade waste heat recovery and heating. This study attempts for the first time to utilize the Co-ah cycle to establish a waste heat recovery system for DCs based on self-owned power plants in steel enterprises, exploring the feasibility of constructing DCs near self-owned power plants and employing the Co-ah cycle for waste heat recovery. The findings of this research can provide valuable references for the future construction and operation of self-operated DCs in Chinese steel enterprises.

## 1 System description

First, the proposed heating system based on Co-ah cycle which used DC waste heat has significant advantages over existing technologies such as traditional boilers, economizers, and heat recovery steam generators. It can use waste heat of DC efficiently and overcome the efficiency bottleneck of general methods in dealing with low-grade heat sources, and further achieves energy savings and carbon footprint reduction of the overall system. In addition, DC can achieve flexible integration with the steel company’s own power plants in the system, reducing overall operating costs and reducing dependence on external energy sources.

**Fig 1:**
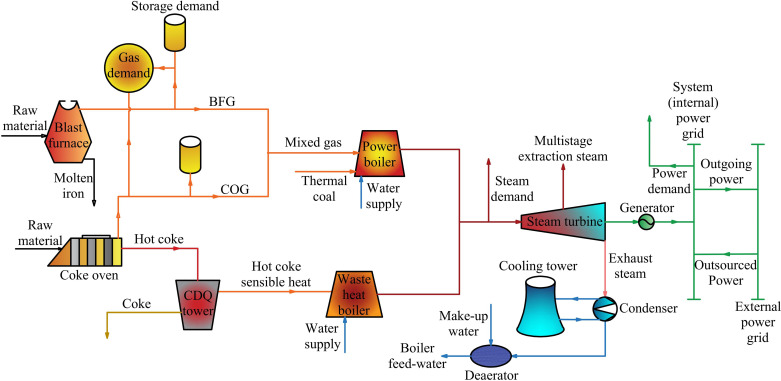
Schematic diagram of self-owned power plant by steel enterprises.

Self-owned power plants in steel enterprises are crucial components of the steam power system, typically consisting of several processes to provide products such as gas, steam, and electricity, as illustrated in Fig 1. The main fuel gases used are Coke Oven Gas (COG) and Blast Furnace Gas (BFG), which are typical byproducts of steel production processes, specifically coking and ironmaking. Due to the relatively low cost of these byproduct secondary energies and optimized large-scale production, the power generation cost of self-owned power plants in steel enterprises is low. Therefore, group-affiliated DCs are benefit from affordable power supply, significantly enhancing their economic viability.

Currently, the primary cost for DCs is electricity, which is almost completely converted into waste heat after use [21]. Utilizing this waste heat for heating is a relatively straightforward waste heat recovery measure. In winter, northern China largely relies on centralized heating. Self-owned power plants can shoulder part of the winter heating load. If the waste heat from newly built or operational DCs in steel enterprises can be integrated into the centralized heating system, it would save a certain amount of heating steam and generate revenue for DC operations. However, the low temperature of existing DC waste heat poses a significant challenge for centralized heating.

The Co-ah cycle is an advanced absorption heating cycle initially designed for recovering waste heat from power plant condensers. Using the Co-ah cycle to recover waste heat from DCs in self-owned power plants of steel enterprises differs from conventional condenser waste heat recovery in general power plants. For typical DC water-cooled backplane systems, the waste heat from chilled water is comparable to the waste heat from circulating cooling water in wet cooling units. However, the waste heat quality is lower but relatively stable, and the quantity of waste heat is related to changes in the IT equipment load inside the DC.

**Fig 2:**
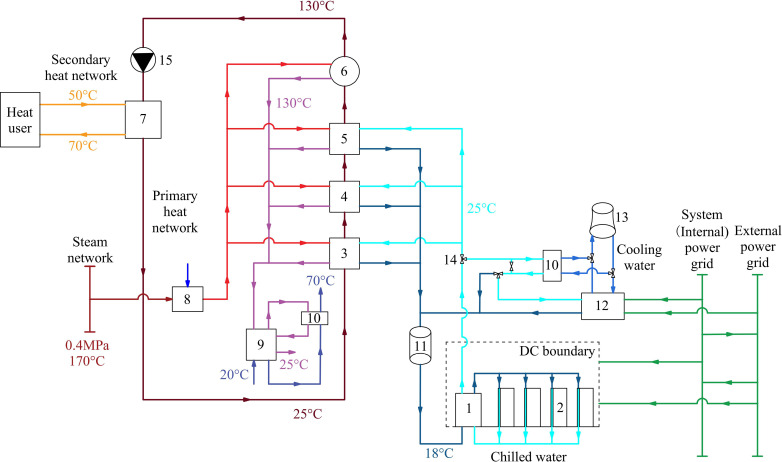
DC waste heat recovery system in central heating by Co-ah cycle.

Additionally, self-owned power plants often consist of multiple boilers and turbines, utilizing various fuels and featuring complex supply and demand scheduling relationships. Therefore, when constructing a Co-ah cycle, it is essential to consider the overall supply and demand scheduling of the self-owned power plant while ensuring load matching between DCs and heat users. The system developed in this study, as shown in Fig 2, will connect to the self-owned power plant and facilitate the trading of steam and electricity. The external power grid will also contribute to some of the system’s electricity demand. The pathways for purchasing electricity primarily depend on the relationship between the internal electricity settlement prices used by the group’s DC enterprises and the time-of-use pricing from the grid.

The system operates mainly in two modes: the heating period and the non-heating period. During the heating period, in addition to electricity, the DC trades some superheated steam with the self-owned power plant to drive the Co-ah cycle. This driving steam serves as a high-temperature heat source. The condensed water generated is circulated through a hot water type heat pump to return to the water supply network of the self-owned power plant at a temperature of about 25°C, while heating the external water supply to about 70°C. This heated water will be supplied to nearby businesses (within 20 km), such as hotels and baths, providing affordable hot water.

Meanwhile, the entire system utilizes the waste heat from the DC as a low-temperature heat source, allowing the DC to cool normally while maintaining the heating supply of the primary network. The return water in the primary network is designed to have temperature rises of 20°C, 20°C, and 15°C through the various stages of heat pumps. Although the steam supply and absorption heat pump units of the self-owned power plant operate stably and reliably, a mechanical refrigeration system is redundantly included for safety in DC cooling.

During the non-heating period, no waste heat recovery occurs. In this case, the chillers and cooling towers operate using fully or partially free cooling methods based on the outdoor Wet Bulb Temperature (WBT).

## 2 System model

### 2.1 Thermodynamic model

The principles of mass conservation and energy conservation based on the first law of thermodynamics are fundamental characteristics of thermal systems. The mass and energy conservation equations under steady-state conditions, as expressed in equations (1) and (2), apply to each component of the system. Additionally, for the self-owned power plant side of the steel enterprise, it is essential to incorporate the boiler efficiency and the turbine consumption characteristics into the energy conservation equations, as referenced in prior research [22].


$$\sum m_{in}=\sum m_{out}$$ (1)

$$\sum m_{in}h_{in}-\sum m_{out}h_{out}=Q-W$$ (2)

In equations (1) and (2), *m* represents the mass flow rate (kg/s), *h* denotes the enthalpy (kJ/kg). *Q* is the thermal power (kW). *W* indicates the work done (kW).

The heat pumps in this system adhere to the thermodynamic relationships of the first type of heat pumps, as expressed in equations (3) and (4). The relationship between the heating coefficient of various heat pumps and the working fluid temperature has been thoroughly investigated in the research [23], adequately meeting the requirements of this study.


$$Q_{A}+Q_{C}=Q_{e}+Q_{g}$$ (3)

$$COP_{h}=(Q_{A}+Q_{C})/Q_{g}$$ (4)

In equations (3) and (4), *Q*_A_, *Q*_C_, *Q*_e_, and *Q*_g_ represent the heat release from the absorber (kW), the heat release from the condenser (kW), the heat absorption by the evaporator (kW), and the heat absorption by the generator (kW), respectively. *COP*_h_ denotes the performance coefficient of the heat pump.

The heating load of the DC can be viewed as the sum of the electrical consumption of all internal equipment except for the chillers, as shown in equations (5) and (6). The power consumption rate of Information Technology (IT) equipment can be calculated using the load factor model developed by Carbó et al. [2,24], and the changes in load factor can be obtained from real DC data [2].


$$P_{DC}=f_{IT}P_{IT,top}+P_{other}$$ (5)

$$f_{IT}=Z_{data}f_{IT,data}+Z_{web}f_{IT,web}+Z_{hpc}f_{IT,hpc}$$ (6)

In equations (5) and (6), *P*_DC_ represents the total electricity consumption of the DC (kW). *f*_IT_ denotes the power consumption rate of IT equipment. *P*_IT,top_ indicates the peak power consumption of IT equipment (kW). *P*_other_ represents the electricity consumption of other equipment (kW). *Z* signifies the proportion of different types of load capacities in the overall DC load capacity. The parameters *f*_IT,data_, *f*_IT,web_, and *f*_IT,hpc_ represent the power consumption rates for data loads, network loads, and computing loads, respectively.

The recommended adjustment method for the Co-ah cycle is that the main network focuses on quality regulation, while the secondary network fully regulates quality [23]. During the actual operation of the Co-ah cycle, if both the primary and secondary networks employ quality regulation, the water temperatures in both networks will decrease synchronously with the decrease of heat load. When the primary network supply water temperature is between 70°C and 130°C, the absorption heat exchanger unit can stabilize the return water temperature of the primary network at approximately 25°C [23]. In the regulation process of the system, the peak heater is given priority, and then each heat pump is adjusted.

### 2.2 Economic analysis model

Analyzing the investment benefits of the system is an important task. This study employs Total Cost of Ownership (TCO) and Net Present Value (NPV) for the analysis [12,25], as represented in equations (7) to (10).


TCO=CAPEX+OPEX+REC−RV (7)

$$NPV=\sum_{i=1}^{N}[CF_{i}/(1+r{)}^{i}]-CAPEX$$ (8)

$$CF_{i}=OPEX_{reference}-(OPEX_{i}-Incomes_{i})$$ (9)

$$NPV{|}_{N=DPBP}=0\to DPBP$$ (10)

In equations (7) to (10), *TCO* represents the Total Cost of Ownership (CNY). *CAPEX* denotes the capital investment cost (CNY). *OPEX* refers to the operating cost (CNY). *REC* indicates the replacement cost (CNY). *RV* represents the residual value (CNY). *NPV* denotes the Net Present Value (CNY). *i* is the calculation year (years). *N* is the calculation termination year (years), typically corresponding to the equipment lifespan. *CF*_i_ denotes the net cash inflow in year *i* (CNY). *r* is the discount rate. *DPBP* represents the payback period for the investment (years).

### 2.3 Energy and environmental benefit evaluation model

The commonly used energy efficiency indicator for DCs, Power Usage Effectiveness (PUE), is typically defined as the ratio of total electricity consumption of the DC to the total electricity consumption of IT equipment. However, PUE alone is insufficient to represent the energy efficiency of a DC with a waste heat recovery system. Therefore, this study employs various energy efficiency indicators suitable for evaluating DC waste heat recovery systems, including *Coal*_save_, *γ*, *PUE’*, *ERE*, *ERF*, and *ExRE.*

*Coal*_save_ indicates the total coal savings of the system (kg), which reflects the coal savings achieved by the DC using this waste heat recovery scheme compared to traditional heating systems and free cooling DCs. This is represented in equation (11).


$$Coal_{save}=m_{sc}[p_{reference}-p_{system}+(Q_{reference}-Q_{system})/(n_{b}q_{c}m_{sc})]$$ (11)

In equation (11), *m*_sc_ represents the coal consumption for power supply (kg/kJ). *P* denotes the electricity consumption (kJ). *Q* indicates the thermal consumption (kJ). “system” refers to the DC and centralized heating system that utilizes this waste heat recovery scheme, while “reference” denotes the isolated traditional heating systems and free cooling DCs. *η*_b_ represents the boiler efficiency, and *q*_c_ signifies the calorific value of standard coal (kJ/kg).

The parameter *γ* represents the effective coefficient of waste heat recovery, indicating the value of waste heat recovery, as shown in equation (12). Waste heat recovery is only meaningful when *γ* is less than 1.


$$\gamma =[P_{system}+Q_{system}/(n_{b}q_{c}m_{sc})]/[P_{reference}+Q_{reference}/(n_{b}q_{c}m_{sc})]$$ (12)

*PUE*’ is an improved energy efficiency indicator specifically designed to evaluate DCs with waste heat recovery systems. It is a modified version of the traditional efficiency indicator *PUE,* which is calculated using *PUE* and *PUE*_wh_, as shown in equation (13). A smaller *PUE’* value indicates higher energy efficiency for the DC.


$$PUE^{\prime }=PUE-PUE_{wh}=P_{DC}/P_{IT}-P_{waste}/P_{IT}$$ (13)

In equation (13), *PUE*_wh_ represents the impact of waste heat recovery benefits on *PUE*, which can be calculated using *P*_waste_ and *P*_IT_ [26]. *P*_IT_ denotes the total electricity consumption of IT equipment (kW), and *P*_waste_ represents the electricity saved due to the waste heat recovery system (kW), expressed as (*Q*_reference_-*Q*_system_)/(*η*_b_·*q*_c_·*m*_sc_) in this study.

Energy Reuse Effectiveness (ERE) and Energy Reuse Factor (ERF) are specific indicators designed to evaluate the utilization capacity of waste heat recovery systems in DCs [27], as shown in equations (14) and (15).


$$ERE=(E_{DC}-E_{reused})/E_{IT}$$ (14)

$$ERF=E_{reused}/E_{DC}$$ (15)

In equations (14) and (15), *E* represents energy consumption (kW), encompassing both electrical and thermal energy, while “reused” refers to energy recovery and utilization. Both indicators can be less than 1. A smaller *ERE* and a larger *ERF* indicate higher energy reuse efficiency of the system.

To avoid a one-sided evaluation of various energy qualities, the Exergy Reuse Effectiveness (ExRE) can also be used for evaluation [28].


$$ExRE=(Ex_{DC}-Ex_{reused})/Ex_{IT}$$ (16)

In equation (16), *Ex* represents exergy (kW).

The environmental benefits of the system can be reflected in the amount of primary energy saved, as shown in equation (17).


$$M_{n}=k_{n}Coal_{save}$$ (17)

In equation (17), *M* represents the emission reduction (kg). *n* denotes the n-th type of pollutant. *k* is the pollutant emission factor per unit of primary energy (kg) [22].

## 3 Case analysis

This study selects Harbin, China, as the research location, using typical meteorological year data for this area. The study chooses Harbin, China as the study site for the case study, mainly because of the unique climatic conditions of the region and the relatively advanced industrial foundation. Harbin is located in a cold northern region with a high-demand for winter heating, making it particularly urgent to improve the energy efficiency of the centralized heating system. In addition, Harbin has many industries, including some important steel enterprises, which provides a good test environment for the practical application of waste heat recovery systems. The parameters related to the DC involved in the study are detailed in Table 1.

**Table 1:** DC parameters.

Item	Description
DC Size	5,250 m² (3.5 m²/ cabinet)
Cabinets	1500 units/ 10 kW per unit
Power Supply Energy Consumption and Loss	15% of IT energy consumption
Lighting and Others	5% of IT energy consumption
Cooling System - Indoor	Water-cooled backplane
Cooling System - Outdoor	Water-side tower free cooling with mechanical refrigeration (Non-heating period) Co-ah Cycle System (Heating period)
Design Chilled Water Supply and Return Temperature	18°C/25°C
Design DC Environment Temperature and Humidity	27°C/60%

**Fig 3:**
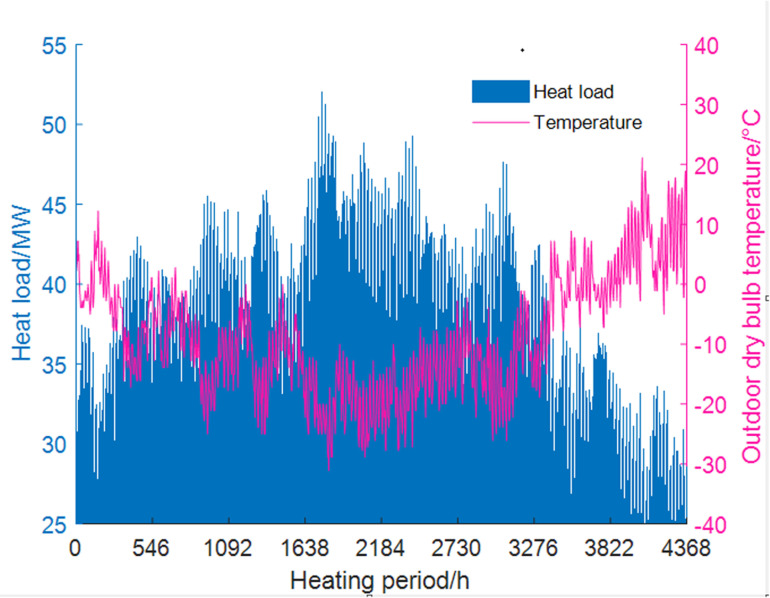
Hourly simulation results of typical residential heating load.

After the waste heat from the DC is integrated into the centralized heating system, it is planned to supply heating and domestic hot water for 14,000 energy-efficient residential areas nearby, which have typical 60 m² apartments and surrounding auxiliary facilities. The eQUEST software is used to simulate the heating duration of 4,368 hours (from October 20th to April 20th). The system’s heat load demand is illustrated in Fig 3.

**Fig 4:**
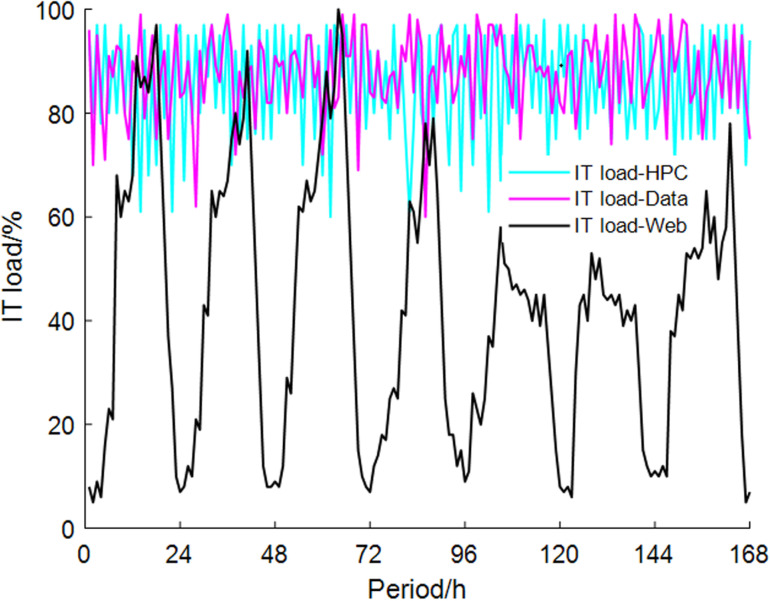
IT equipment load rate change.

**Fig 5:**
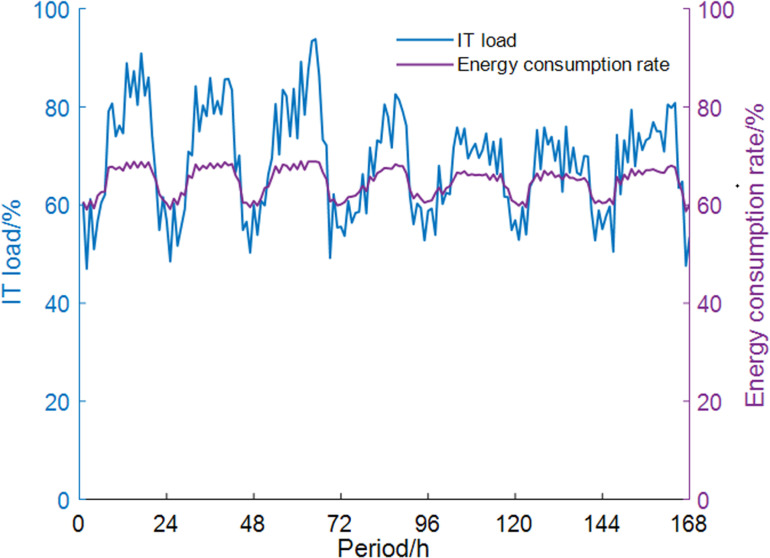
Relationship between DC energy consumption rate and IT equipment load rate.

Since the primary network of the centralized heating system often cannot achieve hourly adjustments [29], the study assumes that the heat load of the primary network is calculated based on the maximum daily demand from heat users. The DC considers a mixed load. Within a typical week, the relationship between load rate and energy consumption rate is shown in Figs 4 and 5. This pattern will be repeated throughout the year.

**Fig 6:**
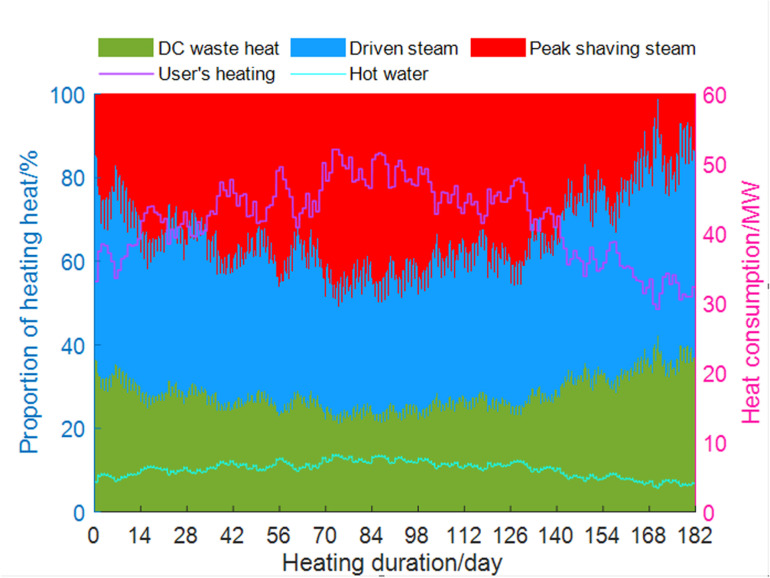
Distribution of system heating load during heating period.

The relationship between the hourly purchased steam heating and the waste heat heating from the DC during the heating period is shown in Fig 6. The figure indicates that under various external thermal loads, the Co-ah cycle can still recover most of the DC waste heat. This system can effectively adapt to load changes between DC and heat users, and may reduce the original steam consumption for heating by about 25%. This suggests that the system not only lowers the cooling electricity consumption of the DC, but may also provide heating benefits.

During the non-heating period, the system operates using a combination of water-side tower **free** cooling and mechanical refrigeration, depending on the external Wet Bulb Temperature (WBT). The conditions for switching between different cooling modes are outlined in Table 2. The COP for the water-cooled chiller is typically set at 6, while the energy consumption of the cooling water pump and cooling tower is approximately 6% and 8% of the energy consumption of the chiller unit, respectively.

**Table 2:** Switching conditions of different cooling modes.

Mode	Requirements
Complete Free Cooling	WBT ≤ 10°C and not in heating period
Mechanical Refrigeration with Free Cooling	10°C < WBT ≤ 15°C and not in heating period
Complete Mechanical Refrigeration	WBT > 15°C and not in heating period
Co-ah Cycle Waste Heat Recovery	During heating period

**Fig 7:**
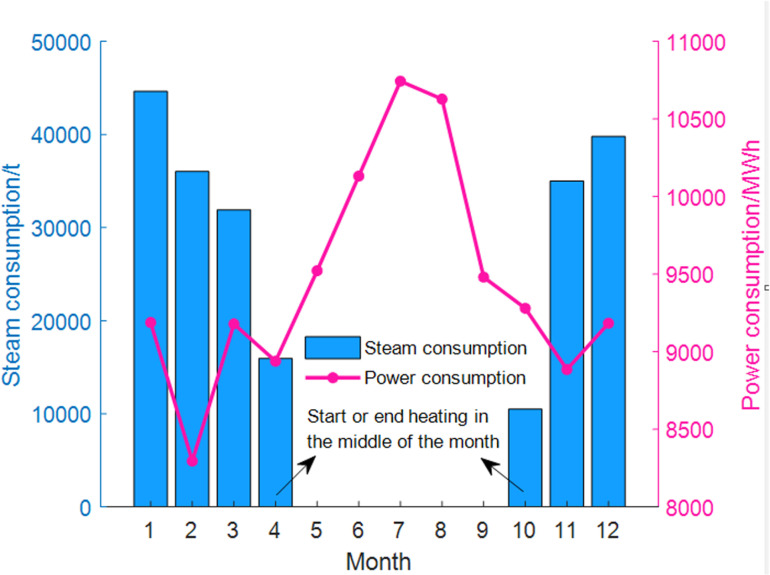
Annual steam and electricity consumption of the system.

The annual electricity consumption and steam consumption of the DC using the waste heat recovery system are illustrated in Fig 7. During the heating period, the substantial input steam not only recovers DC waste heat for heating, but also addresses the cooling issue of the DC, resulting in a significant reduction in system electricity consumption. In the non-heating period, due to the hot summer weather, the system predominantly relies on mechanical refrigeration, leading to higher electricity consumption for the DC during summer.

It is important to note that while the electricity consumption of the system is very low in winter, this comes at the cost of consuming steam. Although the DC waste heat is recovered, the opportunity to utilize free cooling sources is lost. If free cooling is employed year-round, the refrigeration system may not depend on mechanical refrigeration in winter. Therefore, the energy economy and environmental benefits of the system need to be evaluated more comprehensively.

The DC is within the service range of self-owned power plants in steel enterprises, allowing it to choose between external grid electricity or internal power. The external grid electricity price aligns with the large industrial time-of-use electricity prices listed in Table 3, while the internal settlement price is set at 0.40 CNY/kWh. The steam settlement price at 0.4 MPa is 105 CNY/t, which is lower than the steam prices from some CCHP systems [30]. This cost advantage is primarily due to the steel enterprise’s power plant utilizing inexpensive associated secondary energy (BFG/COG) and industrial waste heat to produce steam.

**Table 3:** Electricity price of internal purchase and delivery in iron and steel enterprises.

Item	Time period	Purchase price PriceCNY/kWh	Delivery price PriceCNY/kWh
Peak	8:00-11:00	0.7032	0.3465
	16:00-21:00		
Flat	22:00-6:00	0.5146	0.3465
Valley	6:00-8:00	0.3236	0.3465
	11:00-16:00		
	21:00-22:00		

After purchasing steam from the self-owned power plant, the DC becomes a heat producer and can sell heat, including waste heat, during the heating period. The heat price is associated with the heat supplier, which is the heating company. The heat price of the system is set at 32 CNY/GJ, with hot water priced at 17 CNY/t. The transportation cost is 0.4 CNY/(t·km), and the site water cost is 7 CNY/t. For residents in the Harbin energy-efficient community, heating fees include basic heating fees and metered heating fees. The basic heating fee is 16.54 CNY/m², and the metered heating fee is 44.41 CNY/GJ.

**Fig 8:**
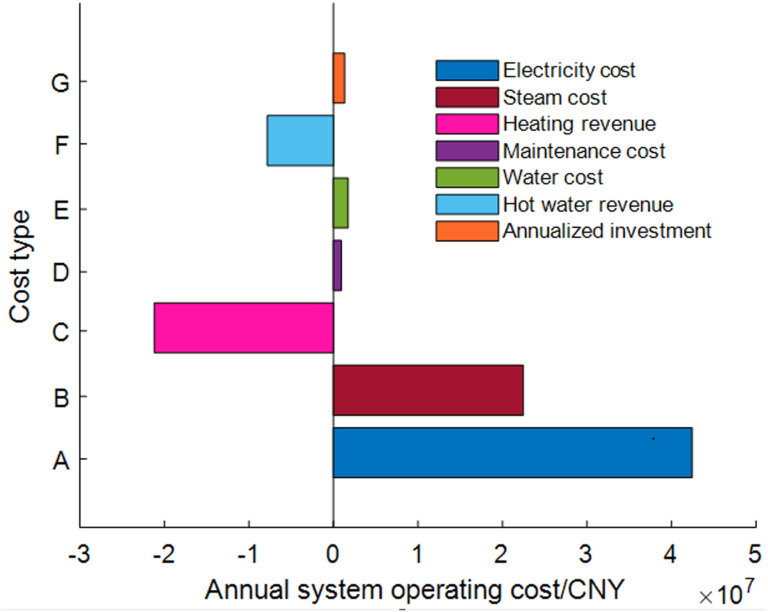
Annual system costs and benefits.

The maintenance and investment costs of the equipment are shown in Table 4, with the installation and construction costs set at 15% of the investment cost. Excluding other uncontrollable costs, the annual costs and revenues of the system are illustrated in Fig 8. The primary cost of the entire system is still electricity purchase, accounting for more than half of the total operating costs. Additionally, the combined revenues from heating and hot water exceed the costs of purchased steam, maintenance, and the annual amortized investment costs. This indicates that the system can generate revenue beyond just reducing cooling electricity costs.

**Table 4:** Incremental investment and maintenance costs.

Equipment	Absorption Heat Pump	Spray Water Cooling	Water-Water Heat Exchanger	Water-Cooled Unit	Steam-Water Heat Exchanger	Chilled/Hot Water Network	Steam Network
Capacity Demand (kW, m)	35,000	1,100	1,700	/	/	2,500	1,000
Investment Unit Price (CNY/kW) or (CNY/m)	400	205	205	/	/	800	1,000
Maintenance Cost (CNY/kWh)	0.0025	0.0022	0.0022	0.0097	0.0022	/	/

The investment mentioned here refers to the incremental investment in this system compared to the water-side tower free cooling system of the combined mechanical refrigeration. It does not include investments in the DC itself, chillers, water-cooled backplane systems, cooling towers, cooling water, or chilled water pumps, as this study considers all comparison objects as the same DC utilizing water-cooled backplane cooling technology. The existing heating pipeline, steam-water heat exchangers, and heating pumps of the self-owned power plant can still be used, and this portion of the investment is not included in the calculation. Moreover, the power cost in the analysis encompasses all necessary electricity consumption for the DC, including IT, power distribution, cooling, and other equipment.

**Fig 9:**
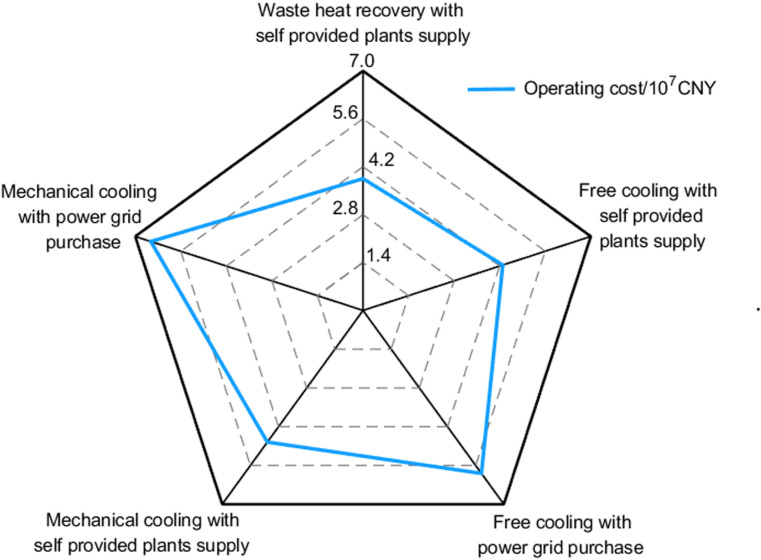
Comparison of annual operating costs of DC in five scenarios.

To analyze the impact of the low electricity prices from self-owned power plants and the proposed waste heat recovery scheme on the overall costs of the **DC**, the operating costs under five different scenarios are compared, as presented in Fig 9. DCs benefiting from self-owned power plants show significant advantages compared to those relying on external grid electricity. DCs operated by steel enterprises in traditional cooling modes are also more cost-effective than those managed by other operators that utilize free cooling technologies.

Under the same pricing and power structure, free cooling incurs lower operating costs than traditional cooling. Furthermore, DCs employing the waste heat recovery system achieve better economic benefits compared to those using free cooling, resulting in annual operating cost savings of 4,435,643 CNY, which represents a 9.7% reduction in original operating costs.

In the economic analysis, NPV and TCO are used to evaluate the advantages and disadvantages of using the proposed waste heat recovery system in the DC compared to using free cooling. The equipment lifespan and assessment period are assumed to be 15 years, with a discount rate set at 10% [31,32]. The tax rate is 10%, and two additional maintenance personnel are considered, with a monthly salary of 12,000 CNY and an annual salary increase of 0.6%. Changes in steam prices, heat prices, and electricity prices over 15 years are difficult to predict, so they are assumed to remain constant.

**Fig 10:**
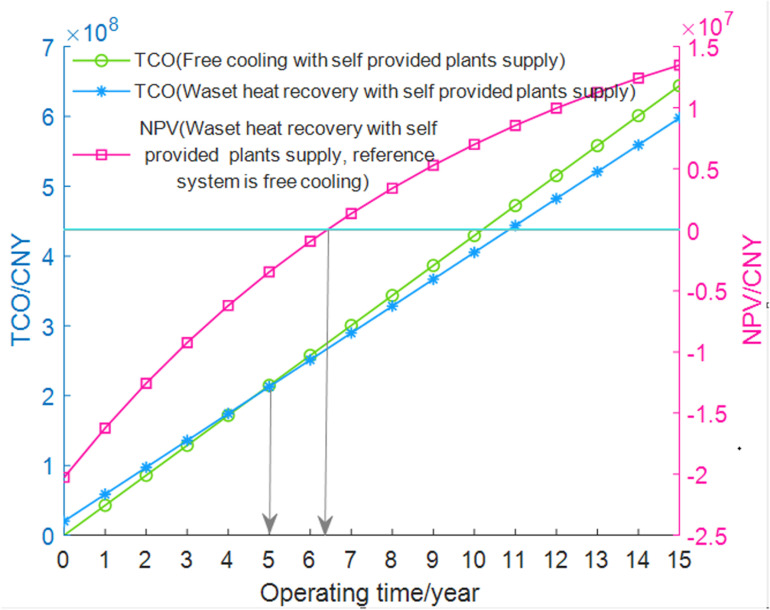
TCO and NPV during the lifespan of DC using waste heat recovery or free cooling.

The results are shown in Fig 10. The NPV calculations indicate that the dynamic payback period for the entire waste heat recovery system compared to the free cooling system is between 6–7 years. After 15 years, the net present value of the system reaches 13,480,606 CNY, which is a good outcome for a large air-cooled DC waste heat recovery system. Additionally, around 5 years into operation, the TCO of this system will be lower than that of the free cooling system, indicating that the waste heat recovery system will be more economical thereafter.

The results of the system efficiency analysis are summarized in Table 5. During the calculations, the boiler efficiency is set at 85%, the standard coal heat value is 29,268.29 kJ/kg, and the coal consumption for electricity supply is 0.312 kg/kWh. The reference temperature for calculating effective energy is based on the average outdoor dry bulb temperature for the typical meteorological year. The results indicate that the heat recovery system, which uses waste heat from the DC for heating, saves 3,671.5 tons of standard coal annually compared to both isolated DC free cooling systems and traditional heating systems. This is due to the simultaneous reduction in both heating coal consumption and cooling electricity consumption. The PUE’ of the system is reduced by 0.146 compared to free cooling, and ERE falls below 1. The ERF indicates that 76.1% of the energy input in the entire DC system is reused.

**Table 5:** Comparison of energy efficiency between DC waste heat recovery and free cooling.

Indicator	System Type	
	Free Cooling with Traditional Heating (Reference System)	Co-ah Cycle Heat Recovery (This Article System)
${Coal}_{save}$	0	3,671.47 t
γ	1	0.941
${PUE}_{wh}$	0	0.138
$PUE^{\prime }$	1.341	1.195
ERE	1.341	0.769
ERF	0	0.761
ExRE	1.341	1.488

However, despite a significant amount of waste heat recovery, the ExRE for the entire year is higher than that of free cooling. This is primarily because the steam consumption of the peak heater is considered a necessary cost for the low-grade waste heat from the DC to be accepted by heat users. Consequently, some inefficiencies from the steam-water heat exchangers are considered in the system. To achieve a lower ExRE value for the DC, it would be necessary to increase the waste heat temperature, such as using a liquid-cooled DC with return water temperatures around 45°C to 60°C. In this case, only minimal or no additional energy input would be required to recover some effective energy.

From an environmental perspective, using this system to recover waste heat from the DC can save primary energy for heating compared to the heat dissipation from free cooling. This can reduce annual emissions by 1,615 tons of CO_2_, 73 tons of SO_2_, 137 tons of NO_X_, 55 tons of particulate matter, and 954 tons of ash, demonstrating significant environmental benefits.

Another important aspect of this study is to evaluate the economic adaptability of self-owned power plants, DC and heating companies to this waste heat recovery system. Economic adaptability refers to whether this system is profitable for the self-owned power plant, DC, and heating company. For the self-owned power plant, it requires economic compensation from the DC for the additional scheduling of steam and electricity and ensures that the operating costs do not exceed those prior to the construction and operation of this system. For the DC, after investing in multi-stage heat pumps and related auxiliary facilities, it is necessary to receive heating and power supply from self-owned power plants to quickly recover costs and profits through heating sales. For the heating company, it requires purchasing heat from the DC at a lower price after constructing the secondary network absorption heat exchangers, enabling investment recovery and profitability.

**Fig 11:**
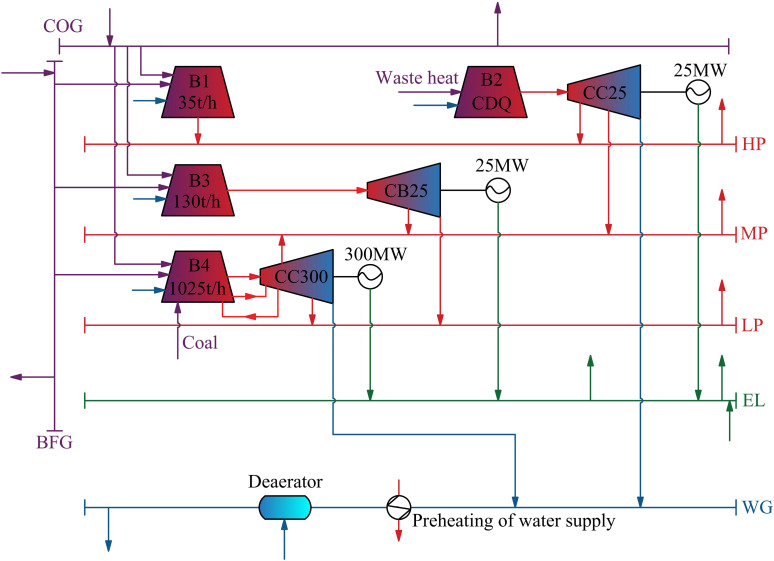
Self-owned power plant system of iron and steel enterprise in this study.

The self-owned power plant studied is shown in Fig 11, where HP, MP, LP, EL, and WG represent the high-pressure, medium-pressure, low-pressure steam networks, internal power grid, and hydraulic pipeline, respectively, with each device having two units. The system itself exhibits varying supply and demand loads and operational parameters over time, which can be scheduled using a minimum operating cost strategy. The connection between the DC and the captive power plant leads to adjustments in supply and demand relationships, thereby altering scheduling strategies and affecting operating costs. To analyze the adaptability of the DC and the self-owned power plant to this waste heat recovery system, an energy scheduling model for the self-owned power plant established in GAMS is employed [22].

**Fig 12:**
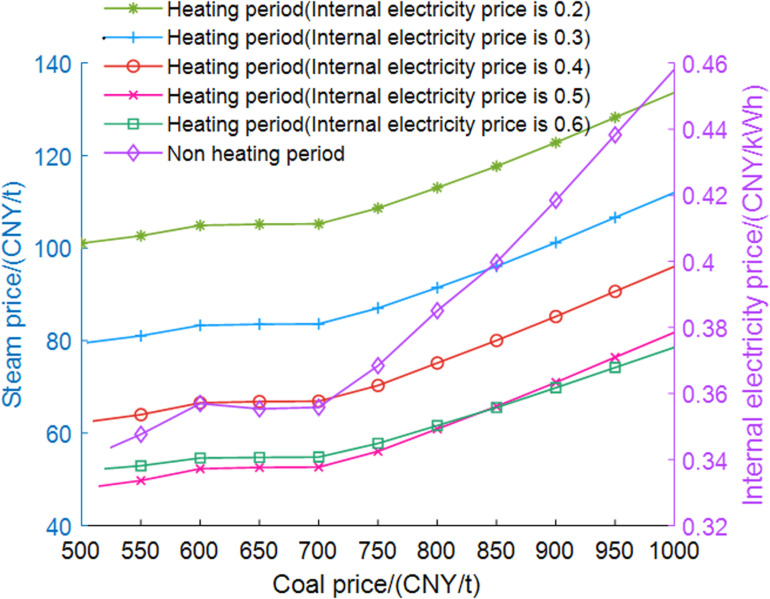
Economic adaptions analysis results of self-owned power plant.

The economic adaptability analysis results of the self-owned power plant are shown in Fig 12. During the heating period, the relationship between five internal settlement electricity prices and the prices of steam and coal is analyzed. During non-heating period, due to the lack of steam demand, the relationship between internal settlement electricity prices and coal prices is provided. Heating period curves represent the selling price of low-pressure steam at which a self-owned power plant maintains constant operating costs under given settlement electricity prices and coal prices. The low-pressure steam price must be above the curve to be favorable for the self-owned power plant. The non-heating period curve is also similar.

As the electricity price sold to the DC increases, the price of steam allowed to be sold to the DC decreases, but the decrease becomes smaller. This is because if the price is lower than the current time-based grid price, DC will only purchase electricity from its own power plants, resulting in a periodic decrease in demand for electricity from its own power plants when the settlement price is too high. Consequently, the growth in revenue for the self-owned power plant becomes less pronounced with increasing electricity prices. If the settlement price is too high, the income may decrease, thereby slowing down the speed of steam price decline.

In the non-heating period, the self-owned power plant will not accept internal settlement electricity prices that fall below the grid electricity export prices or the valley electricity prices, although it may be lower than the normal electricity prices. Additionally, as coal prices rise, the internal settlement electricity prices will also increase.

**Fig 13:**
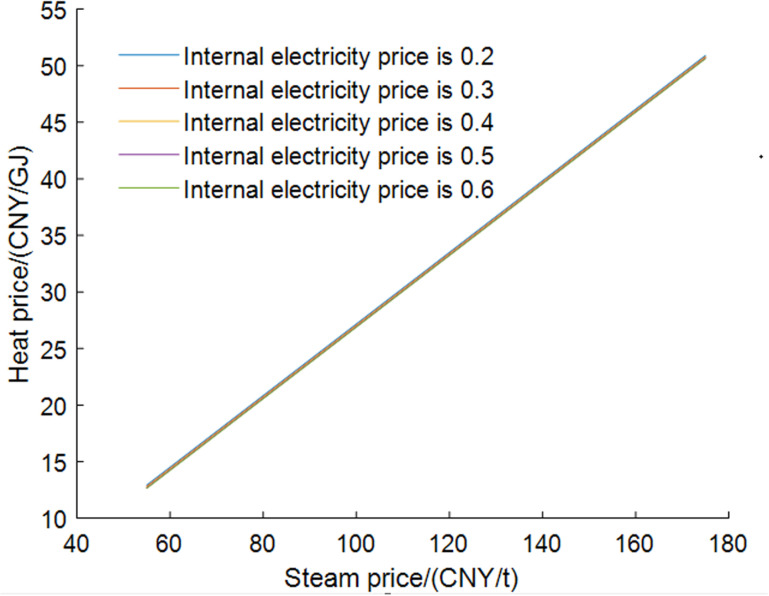
Economic adaptions analysis results of DC.

Fig 13 presents the economic adaptability analysis results for the DC. This indicates that the heating price that DC is willing to accept is linearly related to the purchased steam price, and the favorable heat settlement price is located above the left curve. As the price of purchased steam increases, the accepted heat supply price also rises, but its relationship with the internal settlement electricity price is minimal.

**Fig 14:**
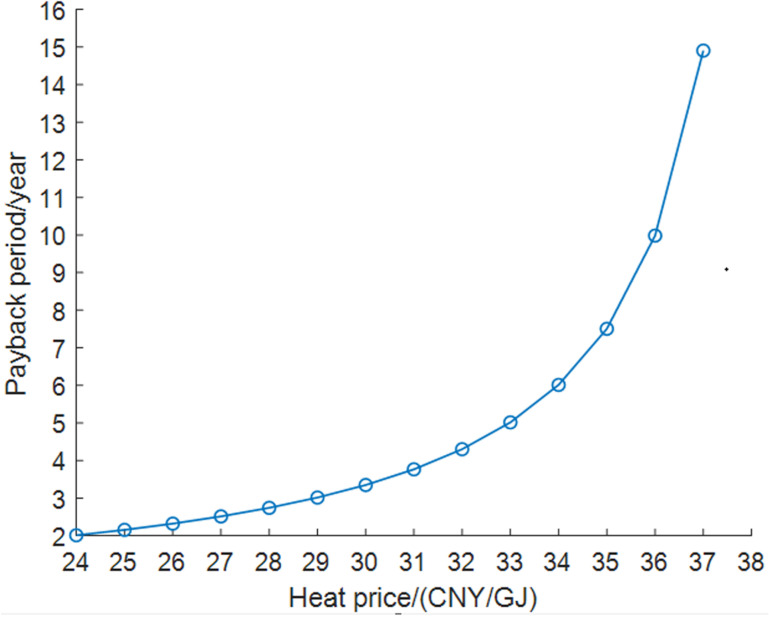
Economic adaptability analysis results of heating company.

For heating companies, the revenue from settling the heat price based on the low-pressure steam price of their own power plants can be used as a reference before recovering waste heat. It is assumed that after the waste heat recovery, the DC may settle with the heating company at a lower heat price. The economic adaptability analysis results are shown in Fig 14.

As the heat price from the DC increases, the payback period for incremental investment in the heating company becomes longer, particularly when the heat price exceeds 34 CNY/GJ.

In this study, the coal price is 750 CNY/t, the steam price is 105 CNY/t, the internal settlement electricity price is 0.4 CNY/kWh, and the heat price is 32 CNY/GJ. There exists a win-win interval for all three parties within the system, indicating that a reasonable energy pricing structure can provide economic benefits to all three stakeholders.

## 4 Discussion

In this paper, a waste heat recovery system based on Co-ah cycle is proposed. By integrating the waste heat from DC with the heat source of the self-owned power plant in iron and steel enterprises, an efficient central heating scheme is formed. The study results show that the system can reduce heating steam consumption by about 25% during the heating period, reduce annual operating costs by 9.7%, reach an annual PUE’ of 1.195, and ERF of 0.761, showing good energy efficiency and economy. From the specific data analysis, the research results reflect the effectiveness and sustainability of the system. During the heating period, the waste heat of the DC is integrated into the central heating system. The results show that the system is able to effectively recover the waste heat generated by the DC while meeting the needs of external heat users. This process not only reduces the steam consumption of traditional heating methods, but also effectively reduces the actual cooling electrical power consumption. The waste heat is converted by a heat pump, allowing the cooling system to meet the heating needs of the main network while maintaining normal operation. Compared to similar studies, Yin et al. proposed a lake water cooling and waste heat recovery integrated system specifically designed for DC. This study evaluates the energy-saving potential of the system and discusses the impact of waste heat recovery location and amount. The results show that waste heat recovery from sources such as chilled water or air in server rooms can significantly reduce the system’s energy consumption. If heat is recovered from the return air of IT equipment, it can reduce system energy consumption by 10.06%. Additionally, by adjusting the temperature of the chilled water supply and air supply, the potential energy savings of the system can reach 26.05% [33]. Monsalves et al. planned a combination configuration of DC flexible cooling equipment and waste heat recovery device based on the Balmorel energy system model, taking Denmark as the research object. The results show that the waste heat recovery technology can replace traditional heat pumps in centralized heating systems. Using this integration scheme can reduce the cost of Denmark’s energy system by 5.1% and carbon emissions by 1.4%. In addition, through further optimization, the cost and carbon emissions can be further reduced, but the specific values are affected by regional characteristics [34]. As mentioned in the introduction, most research on DC waste heat recovery is based on an isolated system without establishing thermoelectric connections with other potential energy systems, which often requires the use of electric heat pumps. This approach is not necessarily economical and reduces the energy-saving potential of the system. The system described in this article is based on the Co-ah cycle, which is an effective strategy for DCs in industrial parks or large industrial projects to build flexible and economical thermoelectric connections with nearby energy systems. In addition, the Co-ah cycle has already had many successful cases of low-grade waste heat recovery in power systems, making it more easily accepted by the industry. The system in this study shows higher economic returns and technical adaptability through comprehensive utilization of low-temperature waste heat, and has a more stable operation mode. In addition, the design of the system allows for the full utilization of the low-cost energy from the self-owned power plant of steel enterprises, which is particularly prominent in the model structure. Technically speaking, the system solution proposed in this study has promising energy-saving and emission reduction benefits. This system reduces the cooling power consumption of DC. In addition, it efficiently recovers DC waste heat, reduces steam consumption for heating, and reduces fossil energy consumption and CO_2_ emissions in two aspects. This meets the development requirements of green industry and green data.

## 5 Conclusions

This study focuses on the DC of steel enterprises and proposes a system that utilizes the Co-ah cycle to recover waste heat from the DC for centralized heating. This system is supported by the enterprise’s self-owned power plant. The results indicate that this system demonstrates strong waste heat recovery capability, saving approximately 25% of the original heating steam during the heating season while meeting the cooling needs of the DC and the heating demands of end users, effectively reducing the cooling electricity consumption of the DC.

Compared to free cooling systems, this system achieves a 9.7% reduction in annual operating costs, with a dynamic payback period of 6–7 years. This waste heat recovery system results in a PUE’ that is 0.146 lower than that of free cooling, and the ERF reaches 76.1%, although the ExRE is slightly higher than that of free cooling.

Through waste heat recovery for heating, the system can save 3,671.47 tons of coal annually and reduce CO_2_ emissions by 1,615 tons, showcasing significant energy and environmental benefits. The waste heat recovery system presents a win-win economic scenario for self-owned power plant, DCs, and heating companies under reasonable energy pricing. The profitability of the self-owned power plant primarily depends on coal prices, internal settlement electricity prices, and steam prices, while the profitability of DCs depends on steam prices and heating supply prices, with less dependence on internal settlement prices. The heating company primarily considers the heat price, which should not exceed 34 CNY/GJ to maintain investment enthusiasm.

Although the waste heat recovery system based on Co-ah cycle has shown significant advantages in terms of energy and environmental benefits, this is still a simulation study for a single region. The economic benefits of the entire system must be reanalyzed locally in the project, as areas with shorter heating time may not be suitable for the system due to the high-investment in heat exchange equipment. In addition, as the equipment is used for a longer period of time, multi-stage heat pumps and other components may require more maintenance, which will significantly increase the maintenance cost of the system. In addition, the operation of the entire system will also be affected by various objective factors, such as changes in environmental parameters, internal and external electricity prices, coal prices, etc., which will affect the economy of the system. Given the limitations mentioned above, future research can be conducted from the following aspects:

(1) Conduct regional adaptability analysis through experiments and further simulations.
(2) Build an Agent using a large model to explore a fast diagnostic method for abnormal operation of multi-stage heat pumps.
(3) Based on the existing system, search for the impact of objective parameter changes on system efficiency, and design the optimal operating strategy of the system through multi-objective optimization methods.

## Supporting information

S1 File The data in Figure 3. (PDF)

S2 File The data in Figure 4. (PDF)

S3 File The data in Figure 5. (PDF)

S4 File The data in Figure 6. (PDF)
