# Supplementary material for: The analysis of waste heat recovery in steel enterprises’ data centers based on the Co-ah cycle
Source: PLoS One. 2025 May 29;20(5):e0323455. doi: 10.1371/journal.pone.0323455 (PMC12121740; doi:10.1371/journal.pone.0323455)
Supplement: S1 File — (PDF) [file pone.0323455.s001.pdf]

The data in Figure 3

Item	Index	Heating period /h							
		~546	1092	1638	2184	2730	3276	3822	4368
Heat load/MW	Mean value	36.3	39.6	40.4	45.0	42.4	40.5	34.7	29.5
	Standard deviation	3.6	3.2	3.1	3.0	3.1	3.0	3.2	2.6
Outdoor dry bulb temperature /C°	Mean value	-4.1	-10.3	-11.8	-20.2	-16.4	-13.5	-2.1	6.6
	Standard deviation	6.2	5.7	5.2	4.4	4.9	5.5	5.6	5.7
