# Supplementary material for: The analysis of waste heat recovery in steel enterprises’ data centers based on the Co-ah cycle
Source: PLoS One. 2025 May 29;20(5):e0323455. doi: 10.1371/journal.pone.0323455 (PMC12121740; doi:10.1371/journal.pone.0323455)
Supplement: S2 File — (PDF) [file pone.0323455.s002.pdf]

The data in Figure 4

Item	Index	Period /h						
		~24	48	72	96	120	144	168
IT load-HPC /%	Mean value	84.8	86.6	86.8	84.6	86.5	86.3	85.7
	Standard deviation	12.2	10.5	11.2	11.0	11.4	8.7	10.2
IT load-Data /%	Mean value	86.1	86.5	88.5	87.1	89.0	88.7	88.1
	Standard deviation	8.5	8.1	7.8	7.7	7.1	7.1	7.6
IT load-Web /%	Mean value	48.4	43.0	50.0	36.2	34.9	32.2	40.6
	Standard deviation	33.2	29.3	31.3	23.9	14.4	16.6	21.2
