# Supplementary material for: The analysis of waste heat recovery in steel enterprises’ data centers based on the Co-ah cycle
Source: PLoS One. 2025 May 29;20(5):e0323455. doi: 10.1371/journal.pone.0323455 (PMC12121740; doi:10.1371/journal.pone.0323455)
Supplement: S3 File — (PDF) [file pone.0323455.s003.pdf]

The data in Figure 5

Item	Index	Period /h						
		~24	48	72	96	120	144	168
IT load /%	Mean value	70.6	69.1	72.6	66.0	66.6	65.4	68.4
	Standard deviation	13.0	12.7	13.1	9.7	6.8	7.5	9.8
Energy consumption rate /%	Mean value	64.9	64.7	65.4	64.1	64.4	64.0	64.9
	Standard deviation	3.5	3.5	3.4	2.9	2.1	2.5	3.0
