# Supplementary material for: The analysis of waste heat recovery in steel enterprises’ data centers based on the Co-ah cycle
Source: PLoS One. 2025 May 29;20(5):e0323455. doi: 10.1371/journal.pone.0323455 (PMC12121740; doi:10.1371/journal.pone.0323455)
Supplement: S4 File — (PDF) [file pone.0323455.s004.pdf]

The data in Figure 6

Item	Index	Heading duration/day						
		~26	52	78	104	130	156	182
DC waste heat /%	Mean value	30.1	27.1	25.1	24.3	26.0	30.2	35.2
	Standard deviation	2.6	1.8	2.0	1.4	1.3	2.8	2.8
Driven steam /%	Mean value	40.4	36.5	33.8	32.6	34.9	40.6	47.3
	Standard deviation	3.5	2.4	2.7	1.8	1.8	3.7	3.8
Peak shaving steam /%	Mean value	29.6	36.4	41.1	43.1	39.1	29.2	17.4
	Standard deviation	6.1	4.1	4.7	3.2	3.1	6.5	6.7
User's heating /MW	Mean value	39.1	43.2	46.7	48.3	45.0	39.0	33.3
	Standard deviation	3.0	2.3	3.3	2.0	1.6	3.3	2.4
Hot water /MW	Mean value	5.5	6.3	7.1	7.4	6.7	5.5	4.4
	Standard deviation	0.6	0.5	0.7	0.4	0.3	0.7	0.5
